# Looking for ugly ducklings: The role of the stability of BrdU-antibody complex and the improved method of the detection of DNA replication

**DOI:** 10.1371/journal.pone.0174893

**Published:** 2017-03-30

**Authors:** Anna Ligasová, Petr Konečný, Ivo Frydrych, Karel Koberna

**Affiliations:** Institute of Molecular and Translational Medicine, Faculty of Medicine and Dentistry, Palacký University in Olomouc, Olomouc, Czech Republic; University College London, UNITED KINGDOM

## Abstract

5-Bromo-2′-deoxyuridine (BrdU) labelling and immunostaining is commonly used for the detection of DNA replication using specific antibodies. Previously, we found that these antibodies significantly differ in their affinity to BrdU. Our present data showed that one of the reasons for the differences in the replication signal is the speed of antibody dissociation. Whereas highly efficient antibodies created stable complexes with BrdU, the low efficiency antibodies were unstable. A substantial loss of the signal occurred within several minutes. The increase of the complex stability can be achieved by *i*) formaldehyde fixation or *ii*) a quick reaction with a secondary antibody. These steps allowed the same or even higher signal/background ratio to be reached as in the highly efficient antibodies. Based on our findings, we optimised an approach for the fully enzymatic detection of BrdU enabling the fast detection of replicational activity without a significant effect on the tested proteins or the fluorescence of the fluorescent proteins. The method was successfully applied for image and flow cytometry. The speed of the method is comparable to the approach based on 5-ethynyl-2′-deoxyuridine. Moreover, in the case of short labelling pulses, the optimised method is even more sensitive. The approach is also applicable for the detection of 5-trifluoromethyl-2'-deoxyuridine.

## Introduction

5-Bromo-2'-deoxyuridine (BrdU) is effectively incorporated into the newly synthesised DNA by cellular DNA polymerases. Therefore, it is often used for the visualisation of cellular replicational activity. For BrdU detection, special antibodies raised against BrdU are necessary. For the effective reaction with incorporated BrdU, those anti-BrdU antibodies require special steps to detect BrdU in DNA as it is hidden in the chromatin structure and is not accessible for an antibody reaction. However, these steps can result in the damage of cellular components [1–7].

The widely-used alternative to BrdU is a method based on 5-ethynyl-2'-deoxyuridine (EdU) [8]. EdU is detected using a click reaction with azide-molecules. The reaction is catalysed by monovalent copper ions [8]. EdU can also be detected using a majority of anti-BrdU antibodies as they cross-react with EdU [9]. Although the method based on EdU is fast and simple, it also has some disadvantages. The main one is the cytotoxicity of EdU, which makes EdU unusable for long-term experiments [10–12]. Another complication is the generation of reactive oxygen species during a click reaction. It negatively affects e.g. the detection of fluorescent proteins. Therefore, special protocols preventing degradation of fluorescent proteins have been suggested [13, 14]. In addition, already submicromolar EdU concentrations affect cell cycle progression. The reason is probably the fact that EdU inhibits thymidylate synthase which leads to an imbalance of the nucleoside and nucleotide pools [10, 12, 15, 16].

Concerning BrdU, a high number of anti-BrdU antibodies is commercially available. We previously showed that they significantly differ in terms of affinity to BrdU, which depends on the BrdU position in the DNA chain. Interestingly, only two of the tested clones were suitable for BrdU detection in all the tested protocols [17].

The widely-used protocols for BrdU detection in DNA are based on the acid treatment where the acid concentration is usually between 1 and 4 M [2, 4, 18]. Such treatment results in depurination and cleavage of the DNA making the BrdU accessible for the reaction with the specific antibodies. Besides the acid treatment, alternative procedures for BrdU detection can be used. They include e.g. enzymatic approaches based on the DNA cleavage with DNA nucleases, alkali treatment with sodium hydroxide based on loosening of DNA structure as a consequence of the deprotonation of the nucleobases or the oxidative degradation of DNA by monovalent copper ions [1, 2, 4, 5]. Probably the one of the potentially best systems is based on the enzymatic treatment by DNase I and exonuclease III [19, 20]. According to our non-published data, the enzymatic approach is strongly dependent on the use of the appropriate anti-BrdU antibodies as well as the fixation used. These are probably the main reasons why this method is far less used than other methods of BrdU revelation. Nevertheless, the enzymatic approach could be, due to the high specificity of enzymes, a very fast variant of BrdU detection with a minimal impact on the cellular structure.

In the present study, we showed that the stability of the BrdU-antibody complex is one of the most critical factors for the successful detection of incorporated BrdU in cellular DNA. The speed of dissociation varied for different anti-BrdU antibodies and depended on the BrdU detection protocol. Our data showed that the stabilisation of the complex can be performed by means of a low concentration of formaldehyde. Although the stabilisation effect was observed also when the quick reaction with the secondary antibody was performed, this form of complex stabilisation is disqualified if centrifugation steps are required. After the stabilisation step, a significant increase in the signal/background ratio occurred in some antibodies exhibiting otherwise low signal/background ratio. In some cases, the increase was so high that it provided the highest signal/background ratio measured in the given BrdU detection protocol. Based on our data, we optimised the approach of fully enzymatic detection of BrdU in formaldehyde- and ethanol-fixed cells. The approach was also optimised with respect to its use for image and flow cytometry. According to our assumption, the optimised method highly preserved the reactivity of the tested proteins with antibodies and did not exhibit a significant impact on the fluorescent proteins.

## Materials and methods

### Cell culture

Human HeLa cells (cervix, adenocarcinoma; a generous gift from Dr. David Staněk, Institute of Molecular Genetics, Prague), HeLa cells stably expressing FUCCI (Fluorescent Ubiquitination-based Cell Cycle Indicator; HeLa-Fucci; a generous gift from Dr. Martin Mistrík, Palacký University, Olomouc) and A549 cells (lung, carcinoma; ATCC) were used in the study. The HeLa cells and HeLa-Fucci were cultivated in Dulbecco’s modified Eagle’s medium (DMEM, Gibco) supplemented with 10% foetal bovine serum (PAA Laboratories), 3.7 g/l of sodium bicarbonate and 50 μg/ml of gentamicin. The A549 cells were cultivated in Ham’s Nutrient Mixture F12 (HyClone) medium supplemented with 10% foetal bovine serum (PAA Laboratories). The cells were cultured in culture flasks or on coverslips (12 mm in diameter) in a Petri dish at 37°C in a humidified atmosphere containing 5% CO_2_.

For the labelling of DNA replication, BrdU was added to the culture medium for 30 minutes (if not stated otherwise). The final concentration of BrdU was 10 μM. If EdU was used, the culture medium was supplemented with 10 μM EdU for 5 or 30 min. The final concentration of 5-trifluoromethyl-2'-deoxyuridine (TFdU) and 5-iodo-2'-deoxyuridine (IdU) was 10 μM. The cells were incubated with IdU or TFdU for 30 min.

The cells were fixed either with 2% formaldehyde for 10 minutes, washed in 1× PBS, permeabilized in 0.2% Triton X-100 for 10 minutes and washed in 1× PBS or were fixed with 70% ethanol at -20°C for at least 1 hr and then washed with 150 mM NaCl and 3 mM KCl.

### BrdU detection

The detection of BrdU by means of monovalent copper ions and exonuclease III or by 2N or 4N HCl were performed according to Ligasová et al., 2012 and Kennedy et al., 2000 [4, 5] with the changes indicated in the text. In some protocols, 40 mM HCl was used instead of 2N or 4N HCl.

### Two-step enzymatic detection

The ethanol-fixed cells were briefly washed with 25 mM Tris-HCl, pH = 7.5 and 150 mM NaCl and 1× buffer for DNase I (10 mM Tris-HCl, pH 7.5, 2.5 mM MgCl_2_, 0.1 mM CaCl_2_). Subsequently, the cells were incubated in a solution consisting of 1× buffer for DNase I, 0.625–5 U/ml DNase I for 30 min at 37°C. After washing, the samples were incubated for 30 min at 37°C in a solution consisting of 1× buffer for exonuclease III (66 mM Tris-HCl, pH 8.0 and 0.66 mM MgCl_2_) and 0.4 U/μl exonuclease III and primary antibody (B44, 0.5 μg/ml) and RNase A (100 μg/ml). After incubation with primary antibodies, the samples were briefly washed and post-fixed with 0.2% formaldehyde for 10 min at room temperature (RT). After washing, the cells were incubated with a secondary antibody solution consisting of 25 mM Tris-HCl, pH = 7.5 and 150 mM NaCl, the secondary antibody conjugated with Alexa Fluor 488 (1:100, Jackson ImmunoResearch) and 10 μM DAPI.

### One-step enzymatic detection

The formaldehyde-fixed and Triton X-100-permeabilized cells or ethanol-fixed cells were briefly washed with 1× buffer for exonuclease III or DNase I (10 mM Tris-HCl, pH 7.5, 2.5 mM MgCl_2_, 0.1 mM CaCl_2_). Then, the cells were incubated in a solution consisting of 1× buffer for exonuclease III, 0.1 mM CaCl_2_, 0.4 U/μl exonuclease III; DNase I (in formaldehyde-fixed cells, the concentration was 20 U/ml; in ethanol-fixed cells, 2 U/ml) and primary antibody raised against BrdU, if not stated otherwise. In some cases, BrdU was detected using a solution composed of 1× buffer for DNase I, DNase I (in formaldehyde-fixed cells, the concentration was 20 U/ml; in ethanol-fixed cells, 2 U/ml) and the primary antibody raised against BrdU.

The primary antibodies in the control experiments were diluted in 25 mM Tris-HCl, pH = 7.5 and 150 mM NaCl. After incubation with solution containing the primary antibodies, the samples were briefly washed and post-fixed with 0.2% formaldehyde or left without a post-fixation. The solution of secondary antibodies was the same as in the case of two-step detection.

The cells were incubated with primary and secondary antibodies for 30 minutes and at 37°C and RT, respectively.

In the co-localization experiments, the primary antibodies against the selected proteins were added to the enzymatic cocktail with anti-BrdU antibody. In these cases, the secondary antibodies were added to the solution of the secondary antibody recognizing the anti-BrdU antibody.

After washing, the samples were mounted in the solution of 90% glycerol, 50 mM Tris-HCl, pH 8.0 and 2.5% 1,4-diazabicyclo[2.2.2]octane and evaluated.

### Antibodies

The following primary antibodies were used: mouse anti-BrdU antibody clones: B44 and 3D4 (BD Biosciences), Bu20a and MoBu-1 (Exbio Praha), BMC9318 (Roche), Bu5.1 (Millipore), Bu6-4 (GeneTex), B33 (Sigma Aldrich) and chicken polyclonal anti-BrdU antibody (Abcam). For the concurrent localisation of DNA synthesis and cellular proteins, we used following antibodies: mouse anti-SC35 antibody (Abcam), rabbit anti-H1.2 antibody (Abcam), rabbit anti-PCNA antibody (proliferating cell nuclear antigen; Abcam), mouse anti-coilin antibody (Abcam), mouse anti-MBD4 antibody (Santa Cruz Biotechnology), rabbit anti-fibrillarin antibody (Abcam) and mouse anti-mitochondrial antibody (MTC02; Abcam).

The following secondary antibodies were used: DyLight 649 anti-mouse, Alexa Fluor^®^ 488 anti-mouse, Alexa Fluor^®^ 488 anti-rabbit and DyLight 649 anti-chicken antibodies (all Jackson ImmunoResearch).

### EdU detection

The incorporated EdU was visualised by a click reaction using Alexa Fluor 488 azide according to the manufacturer’s protocol (30 min, RT, ThermoFisher Scientific). The nuclear DNA was stained by DAPI (10 μM, 30 min, RT).

### Fluorescence microscopy

The images were obtained by an Olympus IX81 microscope (objectives: UPLFLN 10× NA 0.3; LUCPLFLN 20×NA 0.45 and UPLANFLN, 40×, NA 1.3) equipped with a Hamamatsu ORCA II camera with a resolution of 1344×1024 pixels using acquisition software (Olympus).

### Flow cytometry

The A549 cells were labelled with BrdU for 30 min, fixed with 70% ethanol, washed and the incorporated BrdU was detected using a solution containing 1× buffer for exonuclease III, 0.1 mM CaCl_2_, 0.4 U/μl exonuclease III; 2 U/ml DNase I and primary antibody raised against BrdU. The measurements and analyses were conducted on the flow cytometer BD FACSCalibur with a 488-nm 15mW air-cooled argon-ion low-powered laser. As a fluorescence detection system, three PMT detectors (530/30; 585/42; > 670) were used. The DNA synthesis was measured and analysed using BD software CellQuest Analysis Software developed by Verity Software House, Inc. All the measurements were performed for three independent experiments. 10,000 cell nuclei were analysed per experiment.

### Data evaluation

The data were analysed using CellProfiler image analysis software [21, 22] and the final graphs were made in Microsoft Excel. All the measurements were performed for three independent experiments. 10,000 cell nuclei were analysed per experiment. The data are presented as the mean values ± STD.

When the intensity of BrdU or EdU signal was determined, we proceeded as follows if not stated otherwise.

For the comparison of particular samples, we calculated the ratio between the average nuclear signal in replicating and non-replicating cells (**R/non-R ratio**). The fraction of replicating cells was determined by a 30-minute incubation of cells either with BrdU or EdU. The number of replicating cells was determined after the click reaction (in the case of EdU) or after 20-minute incubation in 4N HCl followed by incubation with B44 primary antibody (in the case of BrdU). Both, EdU and BrdU provided similar results. In the HeLa cells, a clear replicational signal was detected in ca 44 ± 6% cells labelled with EdU and ca 46 ± 8% in cells labelled with BrdU. From our experience, an **R/non-R ratio** above 3–4 is sufficient for the clear separation of replicating and non-replicating cells using both systems by CellProfiler image analysis software.

The determination of **R/non-R ratio** was performed similarly as in [17]. Briefly, the images of labelled cells were acquired using Olympus IX81 microscope (objective: UPLFLN 10× NA 0.3) and the BrdU-derived signal in cell nuclei was measured by CellProfiler image analysis software. The nuclei were identified by DAPI staining. For the analysis and calculations, Microsoft Excel was used. For the determination of R/non-R ratio we used the average nuclear signal in the (**F**–0.1) most-labelled nuclei (cells that contain the specific signal = **R**) and the signal in the (0.9–**F**) least-labelled nuclei (cells without any specific signal = **non-R**), where **F** is a fraction of the cells exhibiting signal after the incorporation of BrdU or EdU. For 30-minute labelling time, **F** = 0.44 (see above). For 10,000 evaluated cells per experiment, the number of most-labelled cells was therefore equal to ca 3,400 cells and the number of least-labelled cells to 5,600 cells. In some experiments, the signal intensity was calculated. The signal intensity was equal to the average signal in the (**F**–0.1) most-labelled nuclei.

The average signal of fluorescent proteins was equal to the average nuclear signal.

## Results and discussion

### BrdU and anti-BrdU antibodies create unstable complexes when the protocol based on monovalent copper ions is used

Previously, we described an approach for BrdU detection by means of the oxidative degradation of deoxyribose in a DNA chain. One variant of this technology was based on the combined action of monovalent copper ions and exonuclease III [5]. The approach involves two basic steps. During the first step, the cleavage of BrdU-labelled DNA is performed in the presence of copper(I) ions and oxygen. In the next step, the created gaps are utilized by exonuclease III that degrades DNA strands unmasking BrdU incorporated in DNA. BrdU is then detected by specific antibody. In practice, cells are incubated on the shaker in the solution consisting of 20 mM sodium ascorbate and 40 mM glycine and 8 mM CuSO4 and 200 mM NaCl. After washing with 100 mM Tris-HCl, pH ~7.5, the cells are incubated with the primary antibody supplemented with exonuclease III in 1x buffer for exonuclease III [17].

From our published results, it was obvious that only some of the tested anti-BrdU antibodies are suitable for BrdU detection using monovalent copper ions and exonuclease III [17].

**Fig 1:**
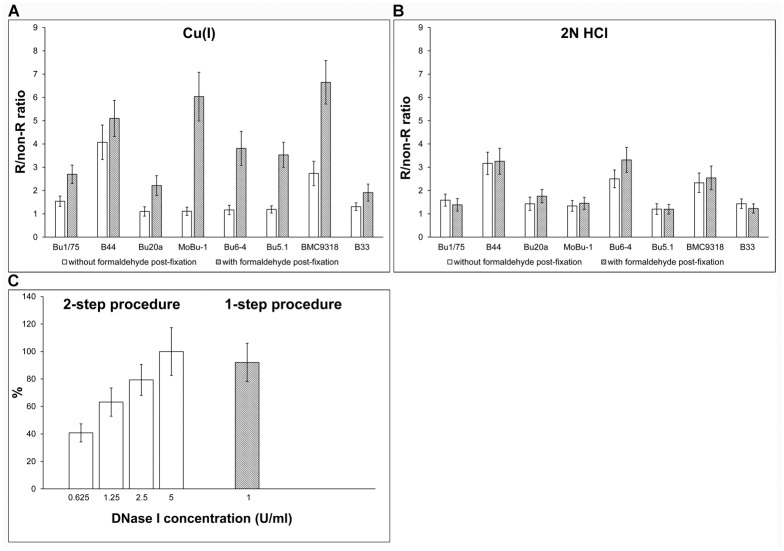
The effect of the stabilisation step and comparison of the one- and two-step enzymatic procedure. **(A)** The effect of the stabilisation step on the BrdU-derived signal in HeLa cells labelled for 30 min with BrdU. The BrdU was revealed by monovalent copper ions and exonuclease III. The data are shown as the mean ± STD. (**B)** The effect of the stabilisation step on the BrdU-derived signal in HeLa cells labelled for 30 min with BrdU. The BrdU was revealed by 2N HCl. The data are shown as the mean ± STD. (**C)** The comparison of the one-step and two-step enzymatic procedures for BrdU detection. Ethanol-fixed HeLa cells were labelled with BrdU for 30 min. The incorporated BrdU was revealed either by incubation with DNase I followed by incubation in the mixture of exonuclease III and the primary antibody (clone B44, two-step procedure) or by incubation of the cells in a mixture of DNase I, exonuclease III and the primary antibody (clone B44, one-step procedure). The R/non-R ratio is normalized to the value for 5 U/ml DNase I concentration equal to 100%. The data are shown as the mean ± STD.

First, we tested if this variability can be accompanied by a different stability of the BrdU-antibody complex. We analysed the BrdU-antibody complex stability in cells incubated for 60 minutes with 10 μM BrdU. The detection of the incorporated BrdU was performed either after the incubation with the monovalent copper ions and exonuclease III or with 2N HCl (Fig 1A and 1B). In these experiments, we incubated cells in a 2% formaldehyde solution in PBS for 10 minutes immediately after the reaction with an anti-BrdU antibody and a 30-second washing step. We supposed that this step could stabilize the DNA-antibody interaction via covalent bonds formation. Formaldehyde was then removed by washing the cells with 25 mM Tris-HCl, pH = 7.5 and 150 mM NaCl and the reaction with a secondary antibody conjugated with fluorochrome followed. Alternatively, the samples were incubated in 1× PBS instead of formaldehyde. We tested 8 different antibody clones raised against BrdU. For the comparison of the particular samples, we calculated the ratio of the average nuclear signal in replicating and non-replicating cells (R/non-R ratio; see Material and methods section). It was obvious that the protocol based on the monovalent copper ions and exonuclease III provided different results for cells with and without the formaldehyde stabilisation step. The signal was higher in formaldehyde-treated samples in all tested antibodies (Fig 1A). On the contrary, the impact of the stabilisation was not significant if BrdU was revealed using 2N HCl (Fig 1B).

It was also clear that, after the copper-exonuclease detection protocol, anti-BrdU antibodies exhibited different dependence on the stabilization step, e.g. in the case of the antibody clone B44, stabilisation with formaldehyde resulted only in a low increase of the R/non-R ratio. On the other hand, in the case of the antibody clone MoBu-1 which exhibited a R/non-R ratio value ca 1 without the stabilization step, post-fixation with formaldehyde resulted in its increase to ca 6.5 (Fig 1A). It was the second highest ratio of all the tested antibody clones. The high increase of the signal was observed also for clones Bu6-4, Bu5.1 and BMC9318. This observation indicated that if the protocol based on monovalent copper ions and exonuclease III is used, the BrdU-antibody complex can quickly dissociate. It seems that the speed of dissociation is inversely proportional to antibody affinity. Concurrently, it is obvious that this effect is not observable in all BrdU detection protocols. We suppose that these differences are caused by the different accessibility of BrdU in DNA chains after the use of various protocols for BrdU revelation and therefore, by the different affinity of antibodies to BrdU in various positions. It is in the complete agreement with our previous findings showing very different affinity of different clones to BrdU at different positions in the oligonucleotide chains [17].

We also analysed the effective concentration of formaldehyde. In this case, BrdU-labelled cells were post-fixed after incubation with a primary antibody alternatively with 0.05, 0.1, 0.2, 0.5, 1 and 2% formaldehyde for 10 minutes and the signal intensity was measured in replicating cells. We normalised the data to the signal obtained from samples post-fixed with 2% formaldehyde (equal to 100%). The average signal intensity in cell nuclei of replicating cells was 82.7±17.2%, 96.7±16.2%, 102.3±15.3%, 95.3±17.3%, 105±18.7% and 100%±12.8% for 0.05, 0.1, 0.2, 0.5, 1 and 2% formaldehyde, respectively. Apparently, the concentrations in the range 0.1–2% were sufficient for effective stabilisation of a BrdU-antibody complex. In further experiments, we post-fixed samples with 0.2% formaldehyde exclusively.

### Optimisation of the enzymatic approach for BrdU detection

Probably the most frequently used approach for the enzymatic detection of incorporated BrdU is the protocol based on DNase I. Already in 1993, Takagi and colleagues improved this protocol. They showed that the use of exonuclease III after the DNase I treatment had a positive effect on the signal value [20]. On the other hand, the use of exonuclease III prolonged the whole procedure. Importantly, they also showed that the results strongly depended on the antibody used. We obtained similar results in our analyses. These two facts probably resulted in the relatively scarce application of DNase I protocols compared to e.g. protocols based on acid treatments.

We optimized the protocol based on DNase I and exonuclease III with respect to its length and sensitivity. First, we tested the possibility of performing the detection of incorporated BrdU in the presence of both enzymes at the same time. We compared the signal strength in samples processed according to the modified previously published protocol—a two-step procedure [20] and our optimised protocol—a one-step procedure (Fig 1C). In accordance with the published protocol, we used cells fixed by ethanol and B44 clone as a primary antibody. According to our previous results this antibody exhibits a very low dependence on the detection protocol [17]. In the case of the one-step procedure, we used 1 U/ml concentration of DNase I and 0.4 U/μl concentration of exonuclease III. If the two-step procedure was performed, we tested concentrations of DNase I in the range of 0.625–5 U/ml, the exonuclease concentration was 0.4 U/μl. In both cases, we post-fixed the antibody by a short incubation of the samples with 0.2% formaldehyde. Our results showed that the optimised one-step procedure provides only a slightly lower signal than the two-step procedure with 5 U/ml DNase I concentration (Fig 1C).

Subsequently, we analysed the impact of the buffer composition and incubation temperature on the signal strength. We found that the buffer for exonuclease III supplemented with calcium ions provided a much higher signal than the buffer recommended for DNase I. Concerning the incubation temperature, 37°C provided higher signal than a temperature of 25°C. The lowering of the temperature could be compensated by the prolongation of the incubation time with enzymes.

**Fig 2:**
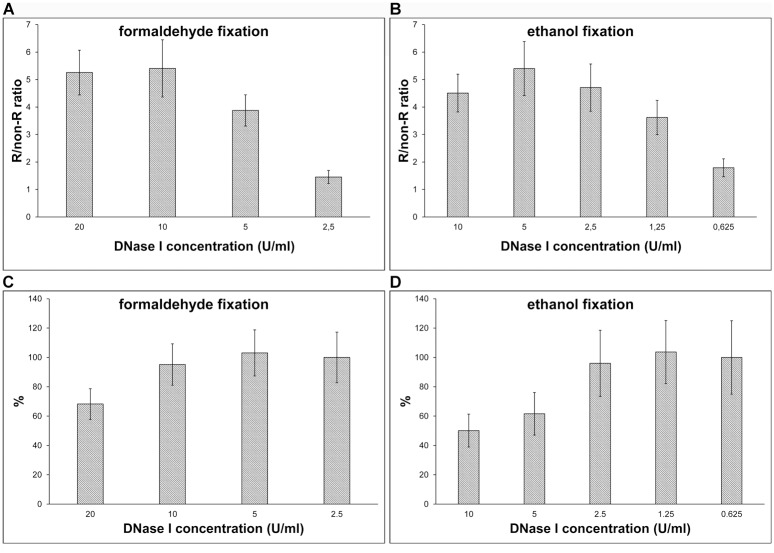
The effect of the DNase I concentration. The effect of the various DNase I concentrations on the BrdU-derived signal and DNA content in formaldehyde- (**A, C**) and ethanol-fixed cells (**B, D**). HeLa cells were labelled for 30 min with BrdU. The BrdU was revealed using various concentrations of DNase I and 0.4 U/μl exonuclease III (**A, B**). The DNA content was determined by the DAPI signal (**C, D**). The data are normalized to the average DAPI signal of 2.5 U/ml (**C**) or 0.625 U/ml DNase I (**D**) concentration equal to 100% in formaldehyde- and ethanol-fixed cells, respectively. The data are shown as the mean ± STD.

We also compared various concentrations of DNase I. In these experiments, both formaldehyde- and ethanol-fixed cells were used (Fig 2). We measured the BrdU-derived (Fig 2A and 2B) and DAPI (Fig 2C and 2D) signals. In the formaldehyde-fixed cells, we tested a DNase I concentration from 2.5 to 20 U/ml (Fig 2A and 2C), in the ethanol-fixed cells from 0.625 to 10 U/ml (Fig 2B and 2D). For BrdU detection, we used the B44 antibody. In the case of formaldehyde-fixed cells, the optimal DNase I concentration was 5–10 U/ml (Fig 2A and 2C), in the case of ethanol-fixed cells the optimal concentration was 1.25–2.5 U/ml (Fig 2B and 2D). The optimal DNase I concentrations provided a relatively high signal without a strong impact on the DNA content measured by DAPI signal. On the other hand, although the concentration of 5 U/ml in ethanol-fixed cells provided the highest signal, this concentration led to a significant decrease of the DAPI signal (Fig 2B and 2D).

### Dissociation of the BrdU-antibody complex plays an important role when BrdU is detected by the enzymatic approach

The optimised protocol is a very fast variant of BrdU detection. However, it was not clear if other anti-BrdU antibodies can be used and eventually what the role of the stability of the BrdU-antibody complex in this protocol is.

**Fig 3:**
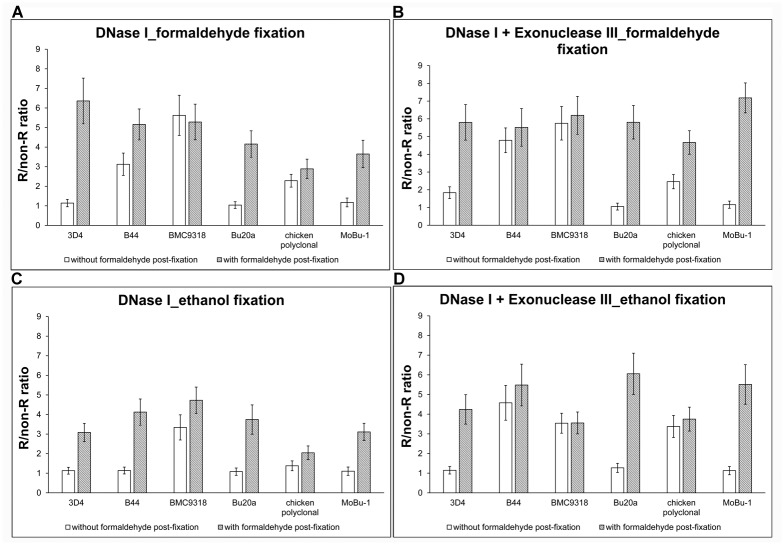
A comparison of signals of different anti-BrdU antibodies using the enzymatic approach and stabilisation step. **(A, B)** The comparison of the BrdU-derived signal in formaldehyde-fixed cells. Six anti-BrdU antibodies were used. The effect of the formaldehyde stabilisation step is shown (grey bars). The incorporated BrdU was revealed either by DNase I alone (**A**) or by DNase I and exonuclease III (**B**). The data are shown as the mean ± STD. (**C, D)** The comparison of the BrdU-derived signal in ethanol-fixed cells. Six anti-BrdU were used. The effect of the formaldehyde stabilisation step is shown (grey bars). The incorporated BrdU was revealed either by DNase I alone (**C**) or by DNase I and exonuclease III (**D**). The data are shown as the mean ± STD.

We analysed the signal of the incorporated BrdU detected by six anti-BrdU antibodies with or without a stabilisation step with the aim to find how significant a role in the signal strength is played by the dissociation of the BrdU-antibody complex. We also compared the signal in cells treated with DNase I alone (Fig 3A and 3C) and with DNase I and exonuclease III (Fig 3B and 3D) in formaldehyde- (Fig 3A and 3B) and ethanol- (Fig 3C and 3D) fixed cells. Our data clearly showed that the stabilisation step is absolutely essential in some cases. In formaldehyde-fixed cells treated with DNase I alone, the post-fixation step led to a significant increase of the signal in all the tested monoclonal antibodies except BMC9318 (Fig 3A). The effect on the polyclonal antibody was not so clear. If formaldehyde-fixed cells were treated with DNase I and exonuclease III (Fig 3B), the highly positive impact of the stabilisation step was observed in the case of 4 antibodies (3D4, Bu20a, MoBu-1 and chicken polyclonal). The impact on B44 and BMC9318 was much lower compared to the rest of antibodies used. When we analysed the ethanol-fixed cells, we found that if the DNase I was used alone (Fig 3C), a positive effect of the stabilisation step was observed in all the tested antibodies. When we used the combination of DNase I and exonuclease III (Fig 3D), the significant positive effect was found in the case of 3 antibodies (3D4, Bu20a, MoBu-1), the effect on the rest of tested antibodies was much lower.

**Fig 4:**
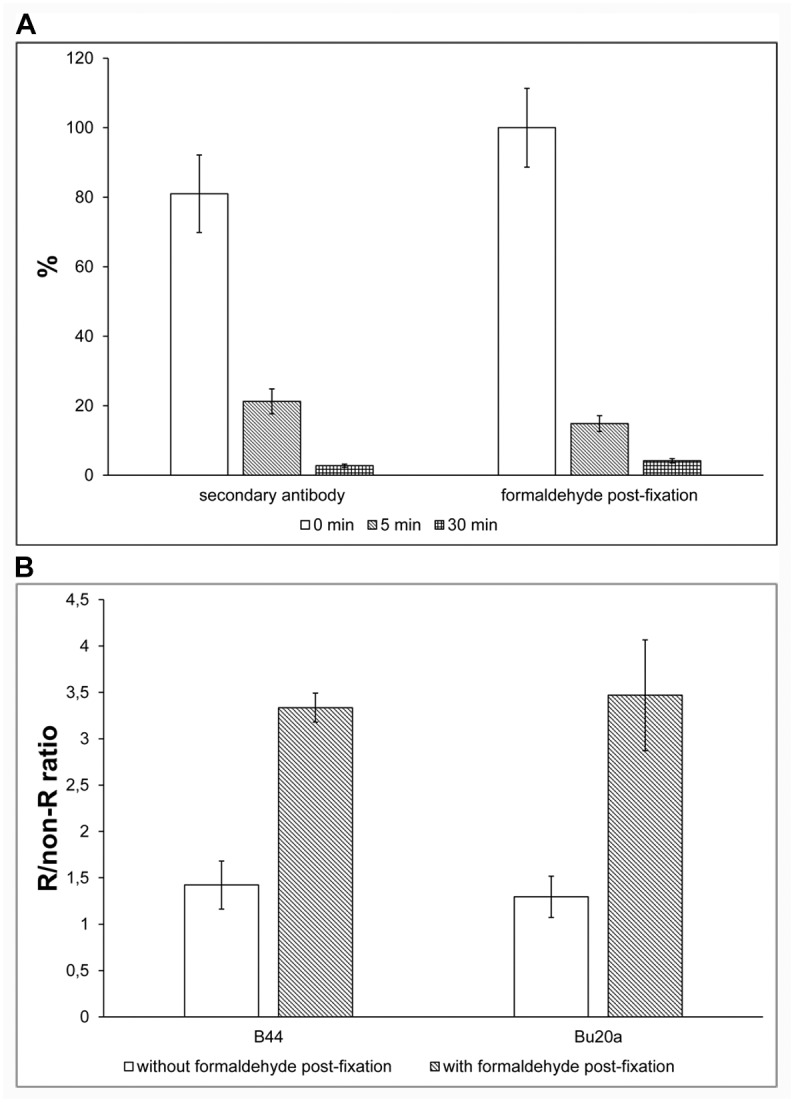
The dissociation of the BrdU-antibody complexes and the impact of the stabilisation on BrdU-derived signal. **(A)** The effect of the length of the washing step after incubation with the anti-BrdU antibody on the BrdU-derived signal. HeLa cells labelled with BrdU were treated with DNase I and exonuclease III and BrdU was detected using Bu20a antibody. After incubation, the samples were washed for 5 s (marked as 0 min in the graph), 5 or 30 min in 1× PBS and then post-fixed with 0.2% formaldehyde or incubated with the secondary antibody. The average nuclear signal in the replicated cells was normalized to the value of the average nuclear signal determined in the sample post-fixed after a 5 s wash in 1× PBS equal to 100%. The data are shown as the mean ± STD. (**B)** The effect of the formaldehyde stabilization on the BrdU-derived signal in HeLa cells after incubation in 40 mM HCl. The BrdU was detected either by B44 or Bu20a antibodies. The effect of the stabilisation step was compared. The data are shown as the mean ± STD.

**Fig 5:**
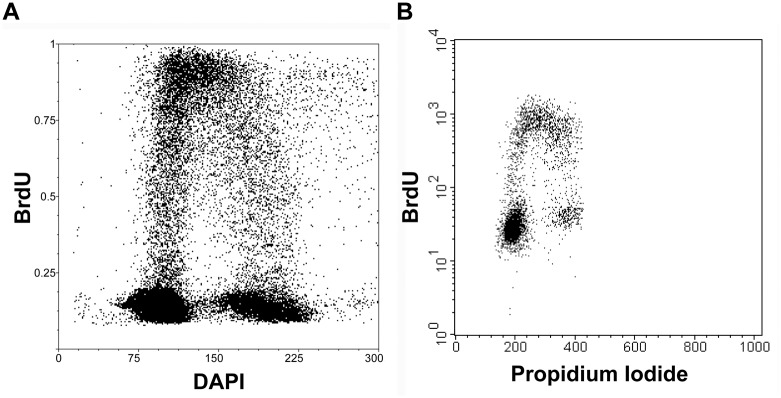
Determination of the cell cycle using image and flow cytometry. **(A)** The bivariate analysis of the replication signal and DNA content in HeLa cells after a 30-minute incubation with 10 μM BrdU using image cytometry. DNase I and exonuclease III were used for BrdU detection in DNA. (**B)** The bivariate analysis of the replication signal and DNA content in A549 cells after a 30-minute incubation with 10 μM BrdU using flow cytometry. DNase I and exonuclease III were used for the BrdU detection in DNA.

We also tested the speed of the dissociation of antibodies from the BrdU epitopes. We incubated HeLa cells with 10 μM BrdU for 30 min and fixed them with formaldehyde. The incorporated BrdU was revealed using DNase I and exonuclease III and detected by the antibody clone Bu20a. Before the incubation with the secondary antibody, we washed the samples for 5 s (time 0 in Fig 4A), or 5 and 30 min with 1× PBS and fixed them with 0.2% formaldehyde for 10 minutes. Alternatively, we did not use a stabilisation step with 0.2% formaldehyde and the cells were incubated with the secondary antibody immediately after a wash with 1× PBS for 5 s or 5 min or 30 min (Fig 4A). It is evident that already a 5-min wash led to an approximately 80% decrease of the signal compared to immediately post-fixed cells. A 30-minute wash step resulted in a nearly zero level of the signal. Only slightly different behaviour was observed, if fixation step was omitted. (Fig 4A). It is evident that the dissociation of the BrdU-antibody complex is a very quick process that can be effectively stopped by post-fixation with formaldehyde or by fast incubation with a secondary antibody.

We also compared the impact of the stabilisation after the quick wash (5 s) by formaldehyde post-fixation or secondary antibody incubation for 3D4 or MoBu-1 antibodies (S1 Fig). It is obvious that the formaldehyde fixation provides better R/non-R ratios than quick incubation with secondary antibody.

Generally, it is clear that the impact of the stabilisation of the BrdU-antibody complex is more significant if DNase I is used alone than if the combination of DNase I and exonuclease III is used. It is also apparent that the stabilisation is a more important prerequisite for a high R/non-R ratio than the use of exonuclease III. Moreover, our results showed that the stabilization has to be performed very quickly as even a several-minute wash can result in a very high decrease of the signal. The stabilization step resulted in better results (higher R/non-R ratio) in the case of some antibodies than in the case of antibodies not requiring stabilisation. It strongly indicates that these “ugly ducklings” exhibit a very high specificity/affinity ratio. It was confirmed by our previous data indicating, that e.g. the Bu20a clone is the clone with the low affinity in most of the tested protocols [17]. Apparently, the stabilisation step preserves the ratio between the signals provided by specifically and non-specifically linked antibodies shortly after the reaction with antibodies. This ratio can make “ugly ducklings” the same or even more attractive than antibodies exhibiting much higher affinity to BrdU.

To exclude the possibility that the low stability of antibodies can be caused by the residual activity of DNase I and/or exonuclease III requiring bivalent cations (Mg^2+^ and/or Ca^2+^) for their action, we added EDTA to the washing solution. The impact of the addition of EDTA was very low if any as compared to the post-fixation with formaldehyde or fast reaction with the secondary antibody. Moreover, and most importantly, the stabilisation effect was observed also in the experiment when we cleaved DNA using a low concentration of HCl without the addition of any enzyme. For example, when we detected incorporated BrdU in ethanol-fixed HeLa cells treated with 40 mM HCl using the B44 or Bu20a antibody, we observed an increase in the signal after the stabilisation with formaldehyde (Fig 4B). Interestingly, if the formaldehyde-fixed cells were used, no such effect was observed. This observation together with the variability of our results for different protocols also clearly showed that antibodies exhibiting high affinity in some protocols require stabilization steps in other protocols.

Although the quick reaction with the secondary antibody can be used instead of formaldehyde fixation, this fast reaction is not possible in some cases. In this respect, we found that the direct addition of formaldehyde to the washing buffer was completely sufficient for the stabilisation of the BrdU-antibody complex without any increase of the background.

### The optimised protocol allows the determination of the cell cycle by image and flow cytometry

The optimised protocol was tested for the determination of the cell cycle using image and flow cytometry (Fig 5, S2 Fig). Both protocols provided sufficient signal after a 30-min labelling of cells with BrdU. Due to the use of the concurrent incubation of anti-BrdU antibody in the solution of DNase I, exonuclease III and eventually also RNase A, it is a very fast variant of BrdU detection.

The optimised protocols for image and flow cytometry are described below:

#### Image cytometry

1. Wash with 1× PBS
2. Fixation
  - ***Formaldehyde fixation***: Fix samples with 1–2% formaldehyde in 1× PBS (10 min, RT), wash with 1× PBS and permeabilise with 0.2% Triton X-100 (10 min, RT). Then, wash samples with 1× PBS (1 hr, RT). Generally, a longer wash step is not critical, but its shortening leads to a decrease of the signal.
  - ***Ethanol fixation***: Fix samples with 70% ethanol (30 min-several weeks, -20°C)
3. BrdU detection
  - Wash with 1× buffer for exonuclease III (3x). According to our observations, one wash is enough.
  - Incubate samples with a mixture of primary anti-BrdU antibody (for the clone B44 the optimal concentration is 0.25–0.5 μg/ml, for Bu20a 2.5–5 μg/ml (for other anti-BrdU antibodies, test the optimal concentration), exonuclease III (0.4 U/μl), DNase I (10 U/ml in formaldehyde-fixed cells, 2–10 U/ml in ethanol-fixed cells), 0.1 mM CaCl_2_ and 1× buffer for exonuclease III (30 min, 37°C). If DNA is labelled using propidium iodide, add RNase A (100 μg/ml) to the mixture.
  - Briefly wash with 1× PBS (2x, together 1 min or less). Incubate with 0.2% formaldehyde in 1× PBS (10 min, RT). It is possible to use a methanol-stabilised stock solution of formaldehyde or a solution prepared from paraformaldehyde in 1× PBS.
  - Wash with 25 mM Tris-HCl, pH = 7.5 and 150 mM NaCl (3x 5 min).
  - Incubate with a secondary antibody and DAPI (10 μM) in 25 mM Tris-HCl, pH = 7.5 and 150 mM NaCl (30 min, RT).
  - Wash in 25 mM Tris-HCl, pH = 7.5 and 150 mM NaCl (3x 5 min).

#### Flow cytometry

1. Centrifuge the samples (500x *g*, 5 min).
2. Discard the supernatant and add 1× PBS (10 ml if 15 ml tubes are used).
3. Centrifuge the samples (500x *g*, 5 min).
4. Discard the supernatant and add 3 ml of 1× PBS. Mix by pipetting.
5. Slowly drop 7 ml of 100% ethanol cooled to -20°C. Put the samples into the freezer and incubate at least for 60 min.
6. Centrifuge the samples (500x *g*, 5 min).
7. Discard the supernatant and add 2 ml of 1× buffer for exonuclease III, mix by pipetting.
8. Centrifuge the samples (500x *g*, 5 min).
9. Discard the supernatant and add 150–200 μl of the mixture composed of exonuclease III (0.4 U/μl), DNase I (2 U/μl) RNase A (100 μg/ml) and the primary antibody (Bu20a, 5 μg/ml) in 1× buffer for exonuclease III and 0.1 mM CaCl_2_ (30 min, 37°C). During incubation, gently shake the tube, do not vortex.
10. Add 3 ml of 1× PBS, mix by pipetting and immediately add 15 μl of 35% stabilised formaldehyde (5 min, RT).
11. Centrifuge the samples (500x *g*, 10 min).
12. Discard the supernatant and add 150 μl of the mixture of the secondary antibody, propidium iodide (10 μg/ml) in 25 mM Tris-HCl, pH = 7.5 and 150 mM NaCl. Mix by pipetting (10x) and incubate for 30 min, RT.
13. Centrifuge the samples (500x *g*, 10 min).

### The optimised protocol allows co-localisation of DNA replication and cellular proteins

**Fig 6:**
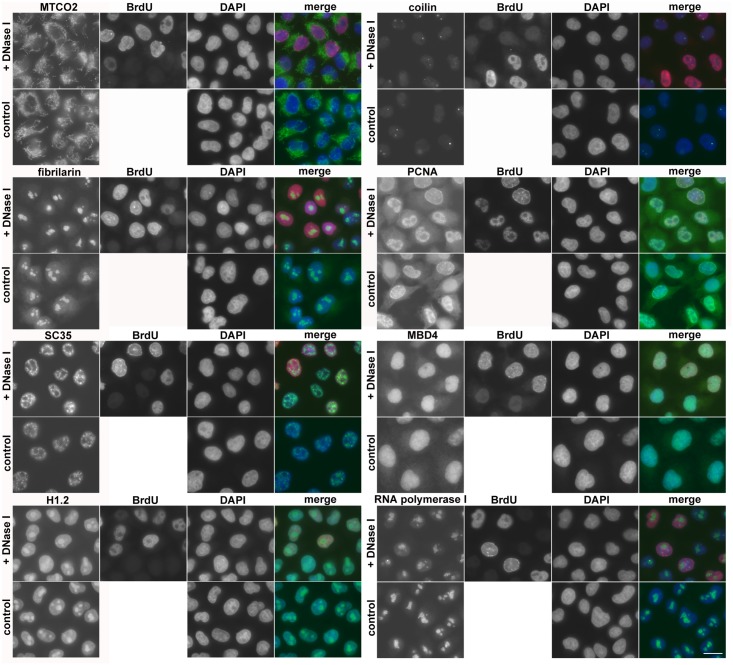
The impact of the optimised protocol on cellular proteins. The co-localisation of DNA synthesis and various cellular proteins is shown. The HeLa cells were labelled with or without (control cells) 10 μM BrdU for 30 min. The cells were incubated with DNase I, exonuclease III, an anti-BrdU antibody and antibody against selected proteins or only with antibody against selected proteins (control cells). Scale bar = 20 μm.

Next, we tested the impact of the optimised approach on the detection of chosen cellular proteins by specific antibodies. We followed these proteins: mitochondrial marker—MTCO2; fibrilarin; sc35; histone H1.2; coilin; PCNA, MBD4 glycosylase and RNA polymerase I subunit—PRAF1 (Fig 6). According to our results, the optimised protocol does not have any significant impact on the tested proteins neither from the point of view of their distribution in the cells nor in terms of the signal strength (Fig 6).

**Fig 7:**
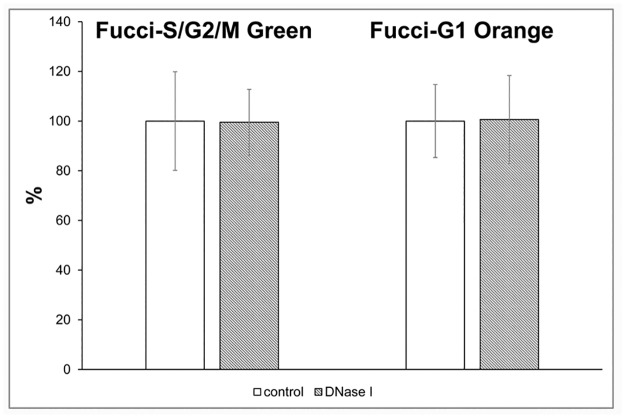
The impact of the optimised protocol on fluorescent proteins. The impact of optimised enzymatic procedure on the fluorescence of HeLa-Fucci cells is shown. The data are normalized to the value of the average signal in the control samples equal to 100%. The data are shown as the mean ± STD.

We also examined the impact of the optimised protocol on the detection of the fluorescent proteins in HeLa-Fucci CCI. We incubated HeLa-Fucci cells for 30 min with 10 μM BrdU, processed them for fluorescence microscopy and analysed the signal of the FUCCI proteins. We did not observe any significant impact of the tested protocol on the signal produced by the fluorescent proteins (Fig 7).

### The optimised method is capable of detecting BrdU after a short labelling pulse and can be used also for other marker nucleosides

**Fig 8:**
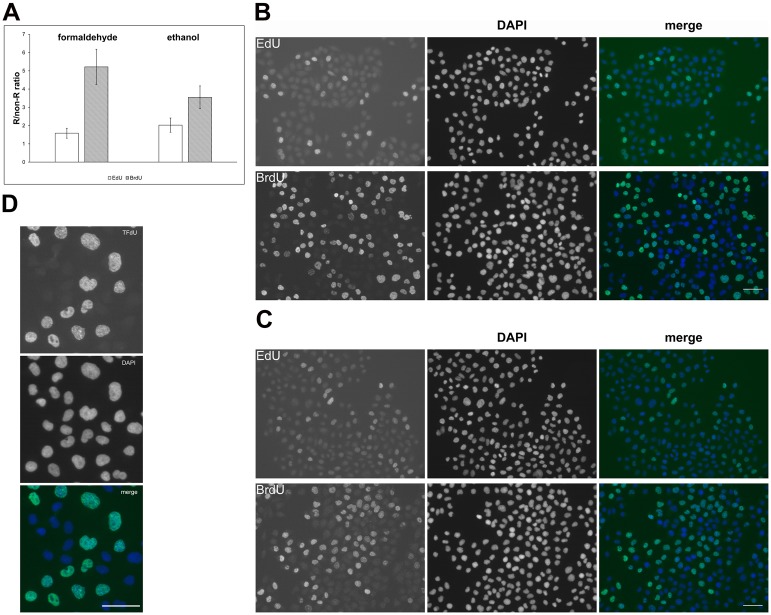
The detection of BrdU and EdU after short labelling pulse and the detection of TFdU. **(A)** The comparison of the R/non-R ratio in formaldehyde- and ethanol-fixed cells labelled for 5 min either with 10 μM BrdU or EdU. The BrdU was detected using the optimised protocol, EdU by click reaction. The data are shown as the mean ± STD. (**B, C)** Examples of microscopic images of cells labelled for 5 minutes with EdU or BrdU and fixed with formaldehyde (B) or ethanol (C). DNA was labelled by DAPI. The scale bar– 50 μm. (**D)** The detection of TFdU in HeLa cells is shown. HeLa cells were labelled with 10 μM TFdU for 30 min and the incorporated TFdU was revealed by optimised protocol. The DNA was labelled by DAPI. The scale bar—50 μm.

As the use of short labelling pulses is necessary for the identification of the nascent DNA chains, we tested the optimised approach of the BrdU detection after a 5-minute labelling pulse. Alternatively, we incubated cells for 5 minutes with 10 μM EdU (Fig 8A–8C). It was obvious that the optimised protocol provides higher sensitivity than EdU, especially in the formaldehyde-fixed cells (Fig 8A–8C). Although the difference was not so expressive in ethanol-fixed cells, the BrdU signal was still 1.7 times higher than the EdU signal.

We also tested the protocol for the detection of IdU and TFdU. IdU-labelled cells provided similar results as BrdU-labelled cells. In the case of TFdU—a part of the TAS-109 drug presently used e.g. for the treatment of some colorectal carcinomas [23], we incubated HeLa cells with 10 μM TFdU for 30 min. It is evident from Fig 8D that the optimised protocol allows the detection of these modified nucleosides as well.

## Conclusions

1. Some protocols for the detection of incorporated BrdU can result in a quick dissociation of the BrdU-antibody complexes.
2. The speed of dissociation depends on the antibody and the protocol used for BrdU detection.
3. The BrdU-antibody complex can be stabilised either by incubation in a formaldehyde solution or by a quick reaction with the secondary antibody.
4. The stabilization step improves the signal/background ratio especially in the case of antibodies exhibiting high specificity/affinity ratio and this ratio can be different in different protocols.
5. The optimised enzymatic protocol
  - provides a high signal/background ratio;
  - has no significant impact on the tested cellular proteins;
  - allows, as compared to EdU, for the reliable analysis of DNA replication when short labelling pulses with a marker nucleoside are used;
  - is compatible with the simultaneous detection of fluorescent proteins;
  - is suitable for image and flow cytometry;
  - allows highly effective detection of other marker nucleosides such as IdU or TFdU.

## Supporting information

S1 Fig The dissociation of the BrdU-antibody complexes in HeLa cells. The effect of the immediate formaldehyde-post-fixation or secondary antibody incubation on the BrdU-derived signal. HeLa cells labelled with BrdU were treated with DNase I and exonuclease III and BrdU was detected using the 3D4 or MoBu-1 antibody. After incubation, the samples were washed for 5 s in 1× PBS and then post-fixed with 0.2% formaldehyde or incubated with the secondary antibody. The average nuclear signal in the replicated cells was normalized to the value of the average nuclear signal determined in the sample post-fixed with formaldehyde equal to 100%. The data are shown as the mean ± STD.
(TIF)

S2 Fig Flow cytometry raw data and example of fluorescence image used in image cytometry analysis. **(A)** Raw data of FSC vs. SSC two-parameter dot-plot picture with cell population used for BrdU vs. PI picture gated is shown. R1 represents the gated cell population used for BrdU vs. PI picture. **(B)** An example of fluorescence image used in the image cytometry analysis. Scale bar 50 = μm.
(TIF)

## References

[1] Ageno M, Dore E, Frontali C. The alkaline denaturation of DNA. Biophysical journal. 1969;9(11): 1281–311. 10.1016/S0006-3495(69)86452-0 4982056 PMC1367631

[2] Dimitrova DS, Berezney R. The spatio-temporal organization of DNA replication sites is identical in primary, immortalized and transformed mammalian cells. Journal of cell science. 2002;115(Pt 21): 4037–51. 12356909 10.1242/jcs.00087

[3] Jackson DA, Pombo A. Replicon clusters are stable units of chromosome structure: evidence that nuclear organization contributes to the efficient activation and propagation of S phase in human cells. The Journal of cell biology. 1998;140(6): 1285–95. 9508763 10.1083/jcb.140.6.1285 PMC2132671

[4] Kennedy BK, Barbie DA, Classon M, Dyson N, Harlow E. Nuclear organization of DNA replication in primary mammalian cells. Genes & development. 2000;14(22): 2855–68. 11090133 10.1101/gad.842600 PMC317063

[5] Ligasova A, Strunin D, Liboska R, Rosenberg I, Koberna K. Atomic scissors: a new method of tracking the 5-bromo-2'-deoxyuridine-labeled DNA in situ. PloS one. 2012;7(12): e52584 10.1371/journal.pone.0052584 23300711 PMC3530445

[6] Stanojcic S, Lemaitre JM, Brodolin K, Danis E, Mechali M. In Xenopus egg extracts, DNA replication initiates preferentially at or near asymmetric AT sequences. Mol Cell Biol. 2008;28(17): 5265–74. 10.1128/MCB.00181-08 18573882 PMC2519731

[7] Tang X, Falls DL, Li X, Lane T, Luskin MB. Antigen-retrieval procedure for bromodeoxyuridine immunolabeling with concurrent labeling of nuclear DNA and antigens damaged by HCl pretreatment. J Neurosci. 2007;27(22): 5837–44. 10.1523/JNEUROSCI.5048-06.2007 17537952 PMC6672250

[8] Salic A, Mitchison TJ. A chemical method for fast and sensitive detection of DNA synthesis in vivo. Proceedings of the National Academy of Sciences of the United States of America. 2008;105(7): 2415–20. 10.1073/pnas.0712168105 18272492 PMC2268151

[9] Liboska R, Ligasova A, Strunin D, Rosenberg I, Koberna K. Most anti-BrdU antibodies react with 2'-deoxy-5-ethynyluridine—the method for the effective suppression of this cross-reactivity. PloS one. 2012;7(12): e51679 10.1371/journal.pone.0051679 23272138 PMC3525573

[10] Cristofoli WA, Wiebe LI, De Clercq E, Andrei G, Snoeck R, Balzarini J, et al 5-alkynyl analogs of arabinouridine and 2'-deoxyuridine: cytostatic activity against herpes simplex virus and varicella-zoster thymidine kinase gene-transfected cells. Journal of medicinal chemistry. 2007;50(12): 2851–7. 10.1021/jm0701472 17518459

[11] Kohlmeier F, Maya-Mendoza A, Jackson DA. EdU induces DNA damage response and cell death in mESC in culture. Chromosome research: an international journal on the molecular, supramolecular and evolutionary aspects of chromosome biology. 2013;21(1): 87–100. 10.1007/s10577-013-9340-5 PMC3601257 23463495

[12] Ligasova A, Strunin D, Friedecky D, Adam T, Koberna K. A fatal combination: a thymidylate synthase inhibitor with DNA damaging activity. PloS one. 2015;10(2): e0117459 10.1371/journal.pone.0117459 25671308 PMC4324964

[13] Hong V, Steinmetz NF, Manchester M, Finn MG. Labeling live cells by copper-catalyzed alkyne—azide click chemistry. Bioconjug Chem. 2010;21(10): 1912–6. 10.1021/bc100272z 20886827 PMC3014321

[14] Loschberger A, Niehorster T, Sauer M. Click chemistry for the conservation of cellular structures and fluorescent proteins: ClickOx. Biotechnol J. 2014;9(5): 693–7. 10.1002/biot.201400026 24639408

[15] Diermeier-Daucher S, Clarke ST, Hill D, Vollmann-Zwerenz A, Bradford JA, Brockhoff G. Cell type specific applicability of 5-ethynyl-2'-deoxyuridine (EdU) for dynamic proliferation assessment in flow cytometry. Cytometry A. 2009;75(6): 535–46. 10.1002/cyto.a.20712 19235202

[16] Ross HH, Rahman M, Levkoff LH, Millette S, Martin-Carreras T, Dunbar EM, et al Ethynyldeoxyuridine (EdU) suppresses in vitro population expansion and in vivo tumor progression of human glioblastoma cells. J Neurooncol. 2011;105(3): 485–98. 10.1007/s11060-011-0621-6 21643840 PMC3202677

[17] Ligasova A, Liboska R, Rosenberg I, Koberna K. The Fingerprint of Anti-Bromodeoxyuridine Antibodies and Its Use for the Assessment of Their Affinity to 5-Bromo-2'-Deoxyuridine in Cellular DNA under Various Conditions. PloS one. 2015;10(7): e0132393 10.1371/journal.pone.0132393 26161977 PMC4498590

[18] Ligasova A, Raska I, Koberna K. Organization of human replicon: singles or zipping couples? J Struct Biol. 2009;165(3): 204–13. 10.1016/j.jsb.2008.11.004 19063972 PMC2670984

[19] Gonchoroff NJ, Greipp PR, Kyle RA, Katzmann JA. A monoclonal antibody reactive with 5-bromo-2-deoxyuridine that does not require DNA denaturation. Cytometry. 1985;6(6): 506–12. 10.1002/cyto.990060604 3905299

[20] Takagi S, McFadden ML, Humphreys RE, Woda BA, Sairenji T. Detection of 5-bromo-2-deoxyuridine (BrdUrd) incorporation with monoclonal anti-BrdUrd antibody after deoxyribonuclease treatment. Cytometry. 1993;14(6): 640–8. 10.1002/cyto.990140608 8404370

[21] Carpenter AE, Jones TR, Lamprecht MR, Clarke C, Kang IH, Friman O, et al CellProfiler: image analysis software for identifying and quantifying cell phenotypes. Genome Biol. 2006;7(10): R100 10.1186/gb-2006-7-10-r100 17076895 PMC1794559

[22] Kamentsky L, Jones TR, Fraser A, Bray MA, Logan DJ, Madden KL, et al Improved structure, function and compatibility for CellProfiler: modular high-throughput image analysis software. Bioinformatics. 2011;27(8): 1179–80. 10.1093/bioinformatics/btr095 21349861 PMC3072555

[23] Kitao H, Morodomi Y, Niimi S, Kiniwa M, Shigeno K, Matsuoka K, et al The antibodies against 5-bromo-2'-deoxyuridine specifically recognize trifluridine incorporated into DNA. Sci Rep. 2016;6: 25286 10.1038/srep25286 27137226 PMC4853717

