# Supplementary figures and images for: Looking for ugly ducklings: The role of the stability of BrdU-antibody complex and the improved method of the detection of DNA replication
Source: PLoS One. 2017 Mar 30;12(3):e0174893. doi: 10.1371/journal.pone.0174893 (PMC5373633; doi:10.1371/journal.pone.0174893)

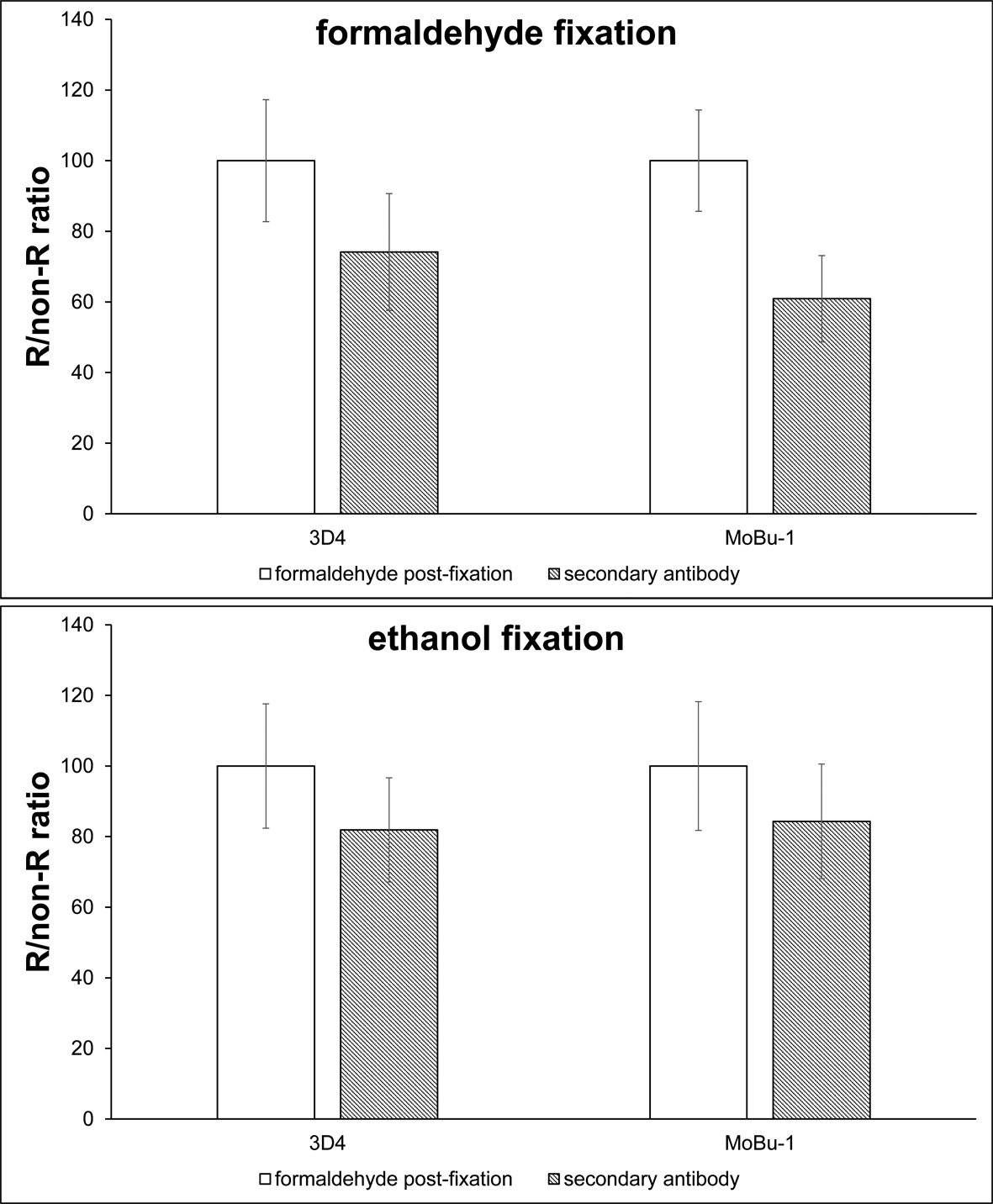

Supplement: S1 Fig — The effect of the immediate formaldehyde-post-fixation or secondary antibody incubation on the BrdU-derived signal. HeLa cells labelled with BrdU were treated with DNase I and exonuclease III and BrdU was detected using the 3D4 or MoBu-1 antibody. After incubation, the samples were washed for 5 s in 1× PBS and then post-fixed with 0.2% formaldehyde or incubated with the secondary antibody. The average nuclear signal in the replicated cells was normalized to the value of the average nuclear signal determined in the sample post-fixed with formaldehyde equal to 100%. The data are shown as the mean ± STD. (TIF) [file pone.0174893.s001.TIF]

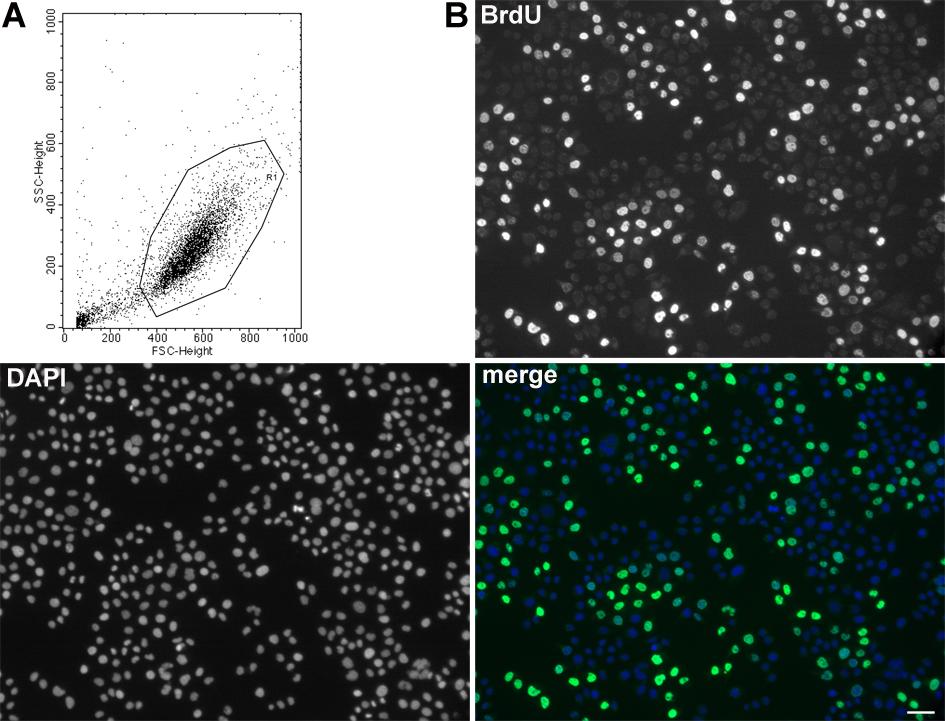

Supplement: S2 Fig — (A) Raw data of FSC vs. SSC two-parameter dot-plot picture with cell population used for BrdU vs. PI picture gated is shown. R1 represents the gated cell population used for BrdU vs. PI picture. (B) An example of fluorescence image used in the image cytometry analysis. Scale bar 50 = μm. (TIF) [file pone.0174893.s002.TIF]
